# Hope and Hopelessness: The Role of Hope in Buffering the Impact of Hopelessness on Suicidal Ideation

**DOI:** 10.1371/journal.pone.0130073

**Published:** 2015-06-24

**Authors:** Jenny M. Y. Huen, Brian Y. T. Ip, Samuel M. Y. Ho, Paul S. F. Yip

**Affiliations:** 1 Hong Kong Jockey Club Centre for Suicide Research and Prevention, University of Hong Kong, Hong Kong Special Administrative Region, China; 2 Department of Psychology, University of Hong Kong, Hong Kong Special Administrative Region, China; 3 Department of Applied Social Sciences, College of Liberal Arts and Social Sciences, City University of Hong Kong, Hong Kong Special Administrative Region, China; 4 Department of Social Work and Social Administration, University of Hong Kong, Hong Kong Special Administrative Region, China; Medical University of Vienna, AUSTRIA

## Abstract

**Objectives:**

The present study investigated whether hope and hopelessness are better conceptualized as a single construct of bipolar spectrum or two distinct constructs and whether hope can moderate the relationship between hopelessness and suicidal ideation.

**Methods:**

Hope, hopelessness, and suicidal ideation were measured in a community sample of 2106 participants through a population-based household survey.

**Results:**

Confirmatory factor analyses showed that a measurement model with separate, correlated second-order factors of hope and hopelessness provided a good fit to the data and was significantly better than that of the model collapsing hope and hopelessness into a single second-order factor. Negative binomial regression showed that hope and hopelessness interacted such that the effect of hopelessness on suicidal ideation was lower in individuals with higher hope than individuals with lower hope.

**Conclusions:**

Hope and hopelessness are two distinct but correlated constructs. Hope can act as a resilience factor that buffers the impact of hopelessness on suicidal ideation. Inducing hope in people may be a promising avenue for suicide prevention.

## Introduction

People react differently to stressors in life, with some individuals deliberately putting an end to their lives in the face of adversity and others endeavouring to proceed. The key to this difference has given rise to suicidology, the scientific study of suicide. Over the last few decades, suicidology has focused on the relationship between various risk factors (in particular negative cognitive factors) and suicide [1–5]. For example, the association between suicidal ideation and psychopathological constructs such as depressive symptoms and hopelessness have been addressed extensively [6–8]. Although the presences of these psychopathological constructs are strong predictors of suicidality, it should not be overlooked that some individuals deal with their hardship in a positive way. The positive elements that motivate people to adopt coping strategies instead of suicidal behaviour in the face of adversity, like any other content associated with a decrease in suicide, may be conceptualized as Papageno effect [9]. Papageno is a character in the famous Mozart’s opera “The Magic Flute” and in one of the scenes Papageno despairs at losing his beloved girl and attempts suicide. His suicidal act is immediately stopped by three child-spirits who later on advise Papageno of a coping strategy. Papageno eventually copes positively with the suicidal crisis after adopting the coping strategy. Niederkrotenthaler and his colleagues [9] studied the associations between media content and suicide rates and they found that coverage on positive coping in adverse circumstances in media reports about suicidal ideation has a Papageno effect and decreases suicide. Existing models of psychopathology and suicidality, with a focus on the association between psychopathological constructs and suicide, cannot account for the Papageno effect exerted by the positive elements which motivate people to face their adverse circumstances in a positive way. This limitation has led to a positive psychology movement for the use of positive psychological constructs in the investigation of psychopathology and suicidality [10–13]. For example, there is on-going interest in incorporating the concept of resilience into the suicidality paradigm [10,14]. Johnson and her colleagues [10] performed an extensive review of 77 suicidality studies that investigated the role of at least one positive psychological construct (a.k.a. resilience factor) in moderating the association between a risk factor and an outcome of suicidality, and introduced a buffering framework to investigate the role of resilience factors (e.g. positive attributional styles & agency) in buffering the impact of risk factors (e.g. hopelessness & perfectionism) on suicidality. Using this framework, the buffering effect of a wide range of positive psychological constructs on suicidal thoughts and behaviours can be explored. Kleiman and his colleagues [15] examined the roles of gratitude and grit as resilience factors against suicidal ideations and they found that gratitude and grit interacted that individuals with higher levels of gratitude and grit at baseline have fewer suicidal ideations over time. In another study [16], Kleiman and his colleagues further provided evidence for the role of gratitude in buffering the association between suicidal ideation and its high risk factors, hopelessness and depressive symptoms. Yet, they did not examine the role of grit in buffering suicide risks associated with hopelessness and depressive symptoms in a similar way. The construct of grit (defined as the extent of perseverance and passion in pursuit of long-term goals [17]) is closely related to the components of hope (consisting of goal-directed determination and planning of ways to meet goals [18]). In the present investigation, a relatively less studied but promising resilience factor based on the adaptive cognitive style of hope will be explored for its buffering impact on suicidal ideation using the buffering framework.

The notion that hope may buffer individuals against suicidal ideation is built on empirical findings in the literature suggesting that hope buffers individuals against psychopathology [19–21] and that hope contributes to better outcomes in a variety of negative situations [18,22–26]. A few studies have considered Snyder’s hope construct within the context of suicidal ideation [27–28] and acquired capability for suicide [29]. The broad benefits of having high level of hope, along with the relevant findings in the literature that hope buffers individuals against psychopathology as well as suicidality, suggest that hope is a promising factor to be examined under the buffering framework for suicidality. According to the theory of hope proposed by Snyder and his fellows [18], low-hope individuals fail to generate alternative pathways either to achieve a blocked goal or to formulate new attainable goals, and thus are prone to suicidal ideation in the face of stressors. However, it is inadequate if we simply test whether high-hope individuals are being associated with lower suicidal ideation than low-hope individuals. As pointed by Johnson and her colleagues [10] in conceptualizing the buffering framework, the validation of proposed resilience factors should go beyond examining the bivariate association between resilience factors and their outcomes. Even though high hope is found to be associated with reduced suicidal ideation, it does not necessarily establish the positive effect of hope as a resilience factor. It may simply demonstrate a reduced risk to its associated risk factor (such as hopelessness) which results in reduced suicidal ideation. In other words, it is not clear whether it is the hope construct or hopelessness construct that should be targeted for research (in predicting suicidal ideation) and psychotherapy (in reducing suicidal ideation). Underlying this problem is a more fundamental question concerning whether hope is simply the inverse of hopelessness, which is a controversial topic to a number of researchers, psychologists and psychiatrists in the field [19, 30–33]. Low hope (characterized by having a lack of positive expectancies for the future) may easily be taken as hopelessness—a state of having increased negative expectancies for the future [34–35]. Hope and hopelessness have been considered to be similar constructs since both tap future-oriented expectancies [36–37], making them appear to be opposite ends of a single bipolar spectrum. However, having increased negative expectancies was not equivalent to having reduced positive expectancies, as illustrated by the findings of a study conducted by MacLeod, Rose, and Williams [38]. In the study, they analysed the patterns of future-orientated expectancies of a group of recent suicide attempters and found that compared with matched controls, recent suicide attempters were having fewer positive future expectancies but no greater negative future expectancies. While these findings suggest that positive future expectancies (encompassed by the hope construct) and negative future expectancies (encompassed by the hopelessness construct) may differ qualitatively, empirical research on the factor structure of both constructs is needed before concluding whether hope and hopelessness constitute opposite ends of a single factor or two separate factors. The present study was conducted for this purpose and the significance of this study lies on an empirical investigation of the two constructs and their relationship to suicidal ideation. This study goes beyond the examination of a direct association between hope and suicidal ideation to investigate hope as a resilience factor which buffers the strength of the association between hopelessness and suicidal ideation. The rationale for this investigation is based on the buffering framework of Johnson and her colleagues [10] which proposes that a resilience factor should be viewed as a separate dimension to the risk factor, and that the resilience factor (i.e., hope in the present study) interacts with the risk factor (i.e., hopelessness in the present study) to reduce the negative impact of the risk factor on an outcome of suicidality (i.e., suicidal ideation in the present study).

In the following sections, the hope construct and the hopelessness construct will be reviewed in their dominant theories (namely Snyder’s theory of hope and Beck’s theory of hopelessness) in the literature, followed by a comparison between the two constructs and an application of the buffering framework in our empirical study to investigate the role of hope in reducing the impact of hopelessness on suicidal ideation.

### Hope under Snyder’s Theory of Hope

In line with the positive psychology movement, Snyder and his colleagues [18] developed a theory of hope (a.k.a. Snyder’s theory of hope) which defines hope as “a cognitive set that is based on a reciprocally derived sense of successful goal-directed determination (termed as *agency*) and planning of ways to meet goals (termed as *pathways*) (p. 571)”. Agency is a sense of determination in achieving goals, which is the motivational component of hope. People with high agency thinking have a strong motivation and great drive to achieve their goals, even when they face difficulties. Pathways refer to one’s ability to generate methods and plans to achieve goals. People with high pathways thinking are more likely to generate more than one pathway to reach a particular goal. Hope is the sum of agency thinking and pathways thinking; both components interact and sustain each other [39].

Snyder [40] pointed out that although previous conceptualizations of hope were goal-directed; they did not detail the cognitive process that reflects one’s hopeful thinking and the means by which goals are pursued. The dual emphasis on the goal itself and the thinking process in pursuit of the goal make Snyder’s theory of hope distinctive from earlier theories of hope [19–21,35]. Most importantly, the connotation of positive expectation of goal attainment in early hope theories has been deemphasized in Snyder’s hope construct. In fact, re-goaling is possible under Snyder’s theory of hope when one fails to achieve the original goal [41].

### Hopelessness under Beck’s Theory of Hopelessness

In 1974, Beck and his colleagues [34] developed a theory of hopelessness to account for depression and they defined hopelessness as the extent of negative attitudes about the future and conceptualized it as the perceptual experience of the anticipation of undesirable situations or consequences that are largely beyond one’s control. Notably, Beck’s theory of hopelessness takes into account of agency-like thoughts, but the idea of goal pursuit is not considered.

Beck’s theory of hopelessness has been applied to understand suicidal behavior [42] and hopelessness has consistently been shown to be one of the best predictors of suicidal ideation and eventual suicide [43–44]. In a 10-year prospective study by Beck and associates [43], it was reported that hopelessness was a strong predictor of future suicide, with ten out of eleven of eventual suicide completers (91%) in a sample of patients with suicidal ideation obtaining high scores on the Beck Hopelessness Scale. Only one completer (9%) had obtained less than the cutoff score (i.e., score of 9). Similar subsequent studies with psychiatric patient samples reported hopelessness to be as high as 90%–94.2% predictive of suicide [43,45]. Beck et al. [45] suggested a sequence of events that eventually leads persons with hopelessness to attempt suicide. These persons misconstrue their experiences in negative ways and anticipate dire outcomes resulting from their problems. They are drawn to suicide as the only way out of their “unsolvable problems” (p.190).

### Hope and Hopelessness

Theoretically, the hope construct (as in Snyder’s theory of hope) and the hopelessness construct (as in Beck’s theory of hopelessness) have different foundations as discussed above. Snyder’s hope construct is a goal-directed model with two cognitive components, agency and pathways, which are the “will” and the “ways” to goal achievement. On the other hand, Beck’s hopelessness construct is an overall negative expectation regarding the future without any consideration of specific goals or their pursuit. In this vein, hopelessness may be more related to the Optimism / Pessimism of Carver’s model [46–48], which are characterized by generalized outcome expectancies (which can be favorable / unfavorable expectations about the future). Snyder’s hope construct is not simply a positive future expectation (c.f. hopelessness as negative future expectation), but also contains a mixture of outcome expectancies (i.e., a sense of agency) and one’s expectancies about whether or not one is able to influence the outcome (i.e., problem-solving abilities). Thus, the hope construct has additive value to the hopelessness construct and they together could serve as a new framework through which outcome variables (such as suicidal ideation in the present study) can be examined.

**Fig 1:**
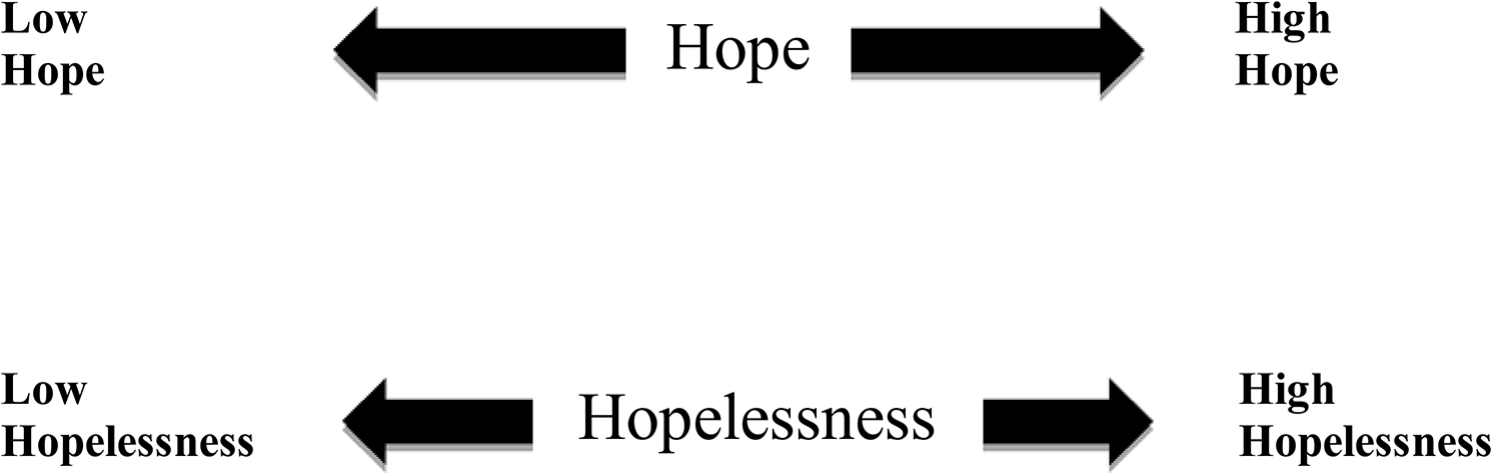
Hope and Hopelessness as Two Separate Constructs.

The concept of hope and hopelessness as two separate constructs is depicted in Fig 1. Instead of being opposite poles of one spectrum, hope and hopelessness have their own bipolar spectra. A person can have a raised sense of agency and problem-solving abilities (i.e., hope) on one spectrum, together with the presence of negative future-oriented thoughts (i.e., hopelessness) on the other. For example, Person A has seasonal affective disorder and he is now having its symptoms during the seasonal change. He oversleeps and overeats, and he has heightened pessimistic feelings about his future. However, along with hopelessness, Person A has some hope as determined by his past successful experiences of coping with seasonal affective disorder and his present attempts to make adjustment to his oversleeping and overeating behaviours and pessimistic thoughts. Indeed, the difference in temporal focus between the two constructs also provides a basis for individuals to have hope and hopelessness at the same time. Whereas hopelessness focuses on the anticipation of future experiences or consequences, hope focuses on past and present experiences of successful goal pursuit.

Other examples of individuals having hope and hopelessness at the same time may come from cancer patients who find ways to make sense out of everyday although they might experience pain, sorrow and sadness. Sullivan [32] did a review on literatures concerning hope and hopelessness at the end of life of these patients and discussed the dynamics of the two constructs during the dying process. He pointed out that hope was still possible at the end of life (a hopeless condition) with varieties of goal: for cure, for survival, for comfort, for dignity, for intimacy or for salvation. If one of the goals could not be met (e.g. hope for survival at the terminal stage of cancer), hope for other goals would emerge. Therefore, the challenge at the end of life may lie on “diversifying and redirecting hope”. The phenomenon of high hope and high hopelessness will be further discussed within the context of suicidal ideation in the following section.

### Moderating Effect of Hope on Hopelessness and Suicidal Ideation

**Fig 2:**
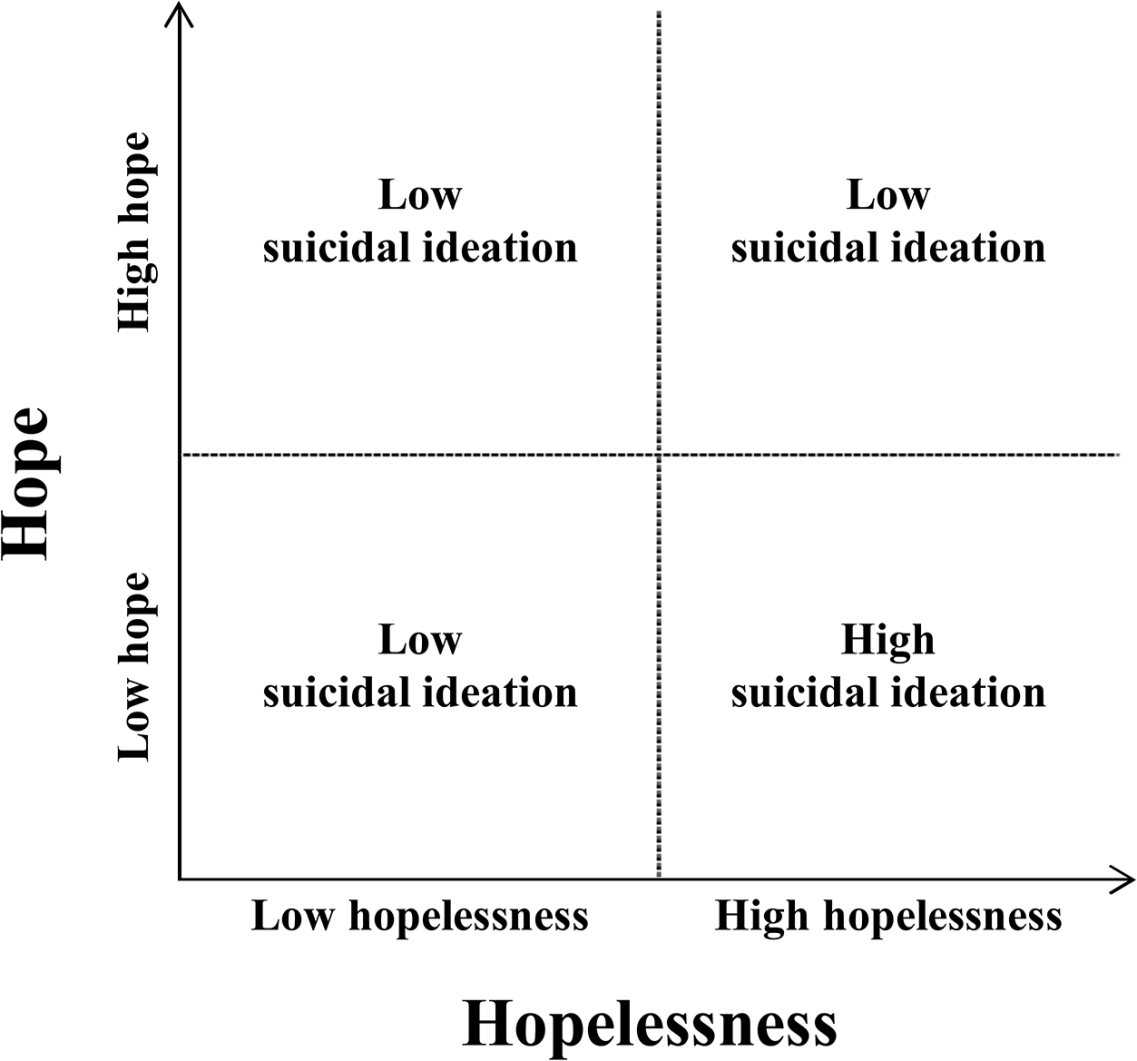
Interaction between Hope and Hopelessness within the Context of Suicidal Ideation.

As hope and hopelessness are two separate constructs, they can interact with each other in four different combinations to result in either high or low likelihood of suicidal ideation as depicted in Fig 2. In particular, the effect of hope in buffering the likelihood of suicidal ideation is evident when individuals are at high levels of hopelessness (i.e., the individuals are at an increased likelihood of suicidal ideation). On the other hand, when the level of hopelessness is low (i.e., the individuals are not at an increased likelihood of suicidal ideation); hope only makes a small difference in the likelihood of suicidal ideation.

The moderating effect of hope on hopelessness and suicidal ideation can be explained by the buffering framework [10] in that a resilience factor (i.e., hope in the present study) interacts with a risk factor (i.e., hopelessness in the present study) to reduce the strength of the association between the risk factor and an outcome of suicidality (i.e., suicidal ideation in the present study). As discussed earlier, previous studies [34,43,45] have shown hopelessness to be strongly associated with suicidal ideation and behaviors. The contemporary view of hopelessness suggests that negative affects embodied by hopelessness may lead to the employment of maladaptive coping styles [49]. A maladaptive coping style, and thus unsuccessful coping with hopelessness, may in turn result in suicidal ideation. At the same time, hope with its equal emphasis on agency and pathways thinking may act on the coping process [50] to buffer individuals at substantial levels of hopelessness against the development of suicidal ideation, or reduce the negative impact of hopelessness on suicidal ideation.

According to Snyder’s theory of hope, suicidal ideation is a result of perceived goal blockage [51]. When a person fails to generate alternative pathways to achieve a blocked goal or formulate new attainable goals, suicidal ideation might arise. As an individual with high hope is better able to generate more strategies for coping with negative stressors, hope is likely to have a moderating effect on the relationship between hopelessness and suicidal ideation. In addition, as high-hope individuals have more goals and can generate more pathways to achieve them, they would be better able to redirect their goals when they encounter goal blockage in adversity. Therefore, hypothetically, under high levels of hopelessness, high-hope individuals would be more likely than low-hope individuals to engage in “re-goaling” and generate adaptive coping strategies to achieve new goals, thus rendering the relationship between hopelessness and suicidal ideation among high-hope individuals comparatively weaker. Hence, high-hope individuals are less likely to develop suicidal ideation even when they are experiencing high levels of hopelessness because they can pursue other goals and rebound from their negative emotional state. Conversely, low-hope individuals who are at high levels of hopelessness are more likely to develop suicidal ideation because of the absence of hope in reducing the negative impact of hopelessness on suicidal ideation.

In summary, the present study made use of empirical data to examine the constructs of hope and hopelessness. It was hypothesized that hope and hopelessness are better fitted as two correlated but distinct factors than one single factor (*H*1). A moderation model of hope buffering the relationship between hopelessness and suicidal ideation was also tested. It was hypothesized that the interaction between hope and hopelessness predicted decreased suicidal ideation (*H*2).

## Methods

Data was collected as part of a population-based household survey in a prevalence study conducted by the Hong Kong Jockey Club Centre for Suicide Research and Prevention of the University of Hong Kong. Ethics approval for the study was granted by the Research Ethics Committee of the Faculty of Social Sciences, the University of Hong Kong. The participants provided their written informed consent to participate in this study. Consent form covering the main points of the study were read to and signed by each participant. Confidentiality of the data was explained to each participant that the information would be used for research purposes only. The participants were informed their rights to terminate the interview survey at any time without any negative consequences. This consent procedure was approved by the Research Ethics Committee stated above.

Respondents were sampled from the Frame of Quarters maintained by the Census and Statistics Department of the Government of the Hong Kong Special Administrative Region. The Frame of Quarters is a complete and up-to-date registry of residential addresses in Hong Kong, which is useful for conducting population-based household surveys. Once a residential address was selected from the Frame of Quarters, a member of that household was randomly selected and invited to respond to the survey. The detailed procedures of the household survey have been reported previously [52–53].

### Participants

Only local residents were eligible to participate in this study. Domestic helpers from overseas countries were excluded from the targeted population. Moreover, only those aged 20 years or above were included in the target population of this study because one of the measures, the Adult Suicidal Ideation Questionnaire (ASIQ [54]), was designed for respondents aged 20 years and above. Participants were being asked their past suicidal behavior and 30 respondents reported having suicide attempt(s) in the year preceding administration of the survey. The data of these respondents with suicide attempt in the year preceding administration of the survey were also included in the present study for they are the population of interest.

Using the above inclusion criteria, a total of 2106 samples were obtained. The response rate was about 62%. The characteristics of the participants (including age group, gender, marital status, employment status and highest educational qualification) are reported in Table 1. The age and gender distribution of our sample was similar to that of and thus representative of the population aged between 20 and 59 years in Hong Kong.

**Table 1:** Characteristics of Participants in the Present Study (Count, Percentage).

Characteristic	Count^a^ (%)	
Age group		
20–29	422	(20.9)
30–39	606	(30.1)
40–49	627	(31.1)
50–59	361	(17.9)
Gender		
Male	955	(47.4)
Female	1061	(52.6)
Marital status		
Single	600	(29.8)
Married	1264	(62.7)
Cohabiting	23	(1.1)
Separated	13	(0.6)
Divorced	78	(3.9)
Widowed	38	(1.9)
Employment status		
Employed on a full-time basis	1274	(63.2)
Employed on a part-time basis	111	(5.5)
Retired / pensioned	54	(2.7)
Home-maker (not otherwise employed)	358	(17.8)
Unemployed	126	(6.3)
Others	5	(0.2)
Not applicable	88	(4.4)
Highest educational qualification		
Tertiary or above	319	(15.8)
Post-secondary	171	(8.5)
Matriculation	111	(5.5)
Vocational qualification (certificate program)	32	(1.6)
Vocational qualification (apprenticeship)	6	(0.3)
IVE program	2	(0.1)
Form 5 graduate	473	(23.5)
High school	158	(7.8)
Junior school	406	(20.1)
Primary school	311	(15.4)
No schooling	17	(0.8)
Unknown / Missing	10	(0.5)

^a^ *N* = 2016

### Measures

Three measures were adapted in the present study: the Hope Scale (Snyder et al. [18]); the Beck Hopelessness Scale (BHS [34]); and the Adult Suicidal Ideation Questionnaire (ASIQ [54]).

#### Hope

Hope was measured by the Hope Scale by Snyder et al. [18]. The Hope Scale is a 12-item, self-reported inventory designed to measure an individual’s level of trait hope (i.e., general or characteristic level of hope across different circumstances). It consists of two subscales: Agency and Pathways. One sample item in the Agency subscale is “I energetically pursue my goals”, and one sample item in the Pathways subscale is “I can think of many ways to get out of a jam”. The original inventory for the Hope Scale [18] contains twelve items in which four items are distracters. For the sake of not making the questionnaire lengthy in the population-based survey, the four distracters were not administered in the present study. A 4-point Likert scale was used, from 1 (definitely false) to 4 (definitely true), with higher scores representing the higher levels of hope. Cronbach’s alpha values for the 12 items in the Hope Scale ranged from .74 to .84; the test-retest correlations ranged from .73 to .85 in different samples [18]. The Chinese version of the Hope Scale from previous studies [22–23] was used in the present investigation.

#### Hopelessness

Hopelessness was measured by the Beck Hopelessness Scale (BHS) by Beck et al. [34]. The BHS consists of 20 statements which measure one’s state of hopelessness in a theoretically-based three-factor structure: feeling about the future (affective component), loss of motivation (motivational component), and future expectations (cognitive component). The internal consistency of the scale has been validated in both clinical and community samples (e.g. KR-20 coefficient = .91 [6]; Cronbach’s α = .87 [55]). The Chinese version of the BHS, translated and validated by Shek [56], was used in the present study. Instead of asking for binary responses (*yes*/*no*) as in the original BHS, which was considered to be a narrow response range [56], the Chinese Hopelessness Scale makes use of the 6-point Likert scale, from 1 (*strongly disagree*) to 6 (*strongly agree*). A higher score in the scale implies a higher level of state hopelessness. The Chinese Hopelessness Scale has also been reported to have good internal consistency (Cronbach’s α = .85) when used in a sample of Chinese college students [56].

#### Suicidal ideation

Suicidal ideation was measured by the Adult Suicidal Ideation Questionnaire (ASIQ) by Reynolds [54]. The ASIQ is a 25-item, self-reported measure of the severity of suicidal ideation in adults. The 7-point scale describes the frequency of the cognitive occurrence of suicidal ideation during the past month, ranging from 0 (*never had the thought*) to 6 (*almost everyday*). A higher score on the scale indicates a higher level of suicidal ideation. The psychometric properties of the ASIQ have been validated in college student samples [9] with good internal consistency (Cronbach’s α = .97) and test-retest reliability (*r* = .86). A Chinese version of the ASIQ has been validated [57], and was used in the present study.

### Statistical Analyses

Two major statistical analyses were conducted to test the two hypotheses in the present study. Confirmatory factor analysis was conducted using LISREL 8.8 to test the hypothesis that hope and hopelessness are better fitted as two correlated but distinct factors than one single factor. Several goodness-of-fit indices were reported: Chi-square goodness-of-fit test statistics, comparative fit index (CFI [58]), incremental fit indices of non-normed fit index (NNFI [59]), and residual based indices of root mean square error of approximation (RMSEA [60]). The following criteria were applied to determine the goodness-of-fit of factor structural models: CFI and NNFI values of 0.90 or greater [58,61], and RMSEA value smaller than 0.08 [62]. For model comparison, chi-square difference test was used to compare nested models; Akaike information criterion (AIC) and consistent Akaike information criterion (CAIC) were considered for non-nested model comparisons. AIC and CAIC measure the parsimonious fit that taking both the model fit and the number of parameter estimated into consideration. Smaller values of AIC and CAIC indicate the model fits better in compromising between the model fit and model complexity.

Negative binomial regression analyses were conducted using IBM SPSS Statistics 20 to test the hypothesis that hope is a moderator in the relationship between hopelessness and suicidal ideation, such that the interaction between hope and hopelessness predicted suicidal ideation. As stated by Gardner, Mulvey, and Shaw [63], negative binomial regression should be used instead of ordinary least squares regression if one or more variables (suicidal ideation in this case) was highly skewed (skew = 6.54, *SE* = .06) in order to avoid the violation of the assumptions of ordinary least squares regression. It should be noted that positive skewness for suicidal ideation was expected for our data because suicidal ideation has a low base-rate of occurrence in the general population. Three negative binomial regression models were fitted to predict suicidal ideation. The first model had the main effect of hopelessness only, and hope was added as another main effect in the second model. The third model had an interaction term of hope and hopelessness in addition to the two main effects. Hope and hopelessness were centered and the interaction term of hope and hopelessness were calculated based on multiplying the centered values of hope and hopelessness in order to avoid possible problems with multi-collinearity. An increase in overall *χ* ^2^ would indicate improved fit of the current model over the previous model. A significant increase in chi-square statistics by analysing the deviance of the negative binomial models would provide further evidence for improved fit over the previous model.

## Results

### Descriptive Statistics and Intercorrelations of Study Variables

The means, standard deviations, Cronbach’s alphas, and intercorrelations of all the study variables in the present study are presented in Table 2. The internal consistency of each of the scales in the present study was estimated using Cronbach’s alpha [64]. The Cronbach’s alphas for Hope Scale, Beck Hopelessness Scale, and ASIQ were .89, .88, and .97 respectively, which were all satisfactory according to the commonly accepted minimum value of .70 [65]. All study variables were significantly correlated in the expected direction. Hope was negatively correlated with hopelessness (*r* = -.54, *p* < .001) and suicidal ideation (*r* = -.25, *p* < .001). Also, hopelessness was positively correlated with suicidal ideation (*r* = .27, *p* < .001). On a related note, the correlations between sub-factors of hope and hopelessness only ranged from-.36 to-.52, indicating that only a variance of 13% to 27% was shared by these two constructs. With little variance in common, hope and hopelessness cannot be considered equivalent constructs.

**Table 2:** Means, Standard Deviations, Cronbach’s Alphas, and Intercorrelations of Study Variables.

Measure	1	2	3	4	5	6	7	8
1. Hope (HS^a^)	.89^d^							
2. Agency	.94^e^	.83^d^						
3. Pathways	.93^e^	.74^e^	.81^d^					
4. Hopelessness (BHS^b^)	-.55^e^	-.55^e^	-.47^e^	.88^d^				
5. Affective	-.54^e^	-.53^e^	-.47^e^	.78^e^	.75^d^			
6. Motivational	-.43^e^	-.43^e^	-.38^e^	.90^e^	.53^e^	.83^d^		
7. Cognitive	-.45^e^	-.47^e^	-.36^e^	.85^e^	.54^e^	.67^e^	.63^d^	
8. Suicidal Ideation (ASIQ^c^)	-.25^e^	-.26^e^	-.20^e^	.28^e^	.22^e^	.24^e^	.25^e^	.97^d^
Mean	24.81	12.10	12.71	54.15	15.51	20.13	18.54	4.37
SD	4.35	2.38	2.28	14.08	4.67	6.96	4.83	11.55

^a^HS = Hope Scale

^b^BHS = Beck Hopelessness Scale

^c^ASIQ = Adult Suicidal Ideation Questionnaire.

^d^Cronbach’s alphas are on diagonal.

^e^All correlations with *p* < .001.

### Confirmatory Factor Analysis on the Constructs of Hope and Hopelessness

Confirmatory factor analysis was conducted to test whether hope and hopelessness fit better into a model as a single construct (manifested as two opposite directions of one construct) or two separate constructs (manifested as two distinct but correlated constructs). The factor structures of hope and hopelessness were tested. The two-factor model (Agency and Pathways), as proposed by the Hope Scale [18], provided a good fit to the data, χ^2^ (19, *n* = 2001) = 553.22, *p* < .001; CFI = .96; NNFI = .95. The three-factor model (Affective, Motivational and Cognitive components), as proposed by the Beck Hopelessness Scale [34], also provided a good fit to the data, χ^2^ (167, *n* = 1953) = 2317.71, *p* < .001; CFI = .94; NNFI = .94. Two sets of alternative measurement models which manifested different combinations between the construct of hope (8 items in total) and hopelessness (20 items in total) were proposed at first-order and second-order levels. The first set of measurement models evaluated was first-order factor models: a one-factor model and a two-factor model. The one-factor model assumes all 28 items (8 items from the Hope Scale and 20 items from the Beck Hopelessness Scale) reflect a single dimensional factor, while the two-factor model assumes that two correlated dimensions (Hope and Hopelessness) underlie the responses to the 28 items. The other set of measurement models being evaluated were second-order factor models: A single second-order factor model and a two second-order factor model. The single second-order factor model is a higher-order model in which a single second-order factor (named as Future Oriented Cognition) underlies the covariation among the five first-order latent factors of hope (Agency and Pathways) and hopelessness (Affective, Motivational and Cognitive components). The two second-order factor model is a higher-order model consisting of two negatively correlated second-order factors (Hope and Hopelessness). The first of these underlies the covariation between the first-order factors (Agency and Pathways), and the second underlies the covariation between the first-order factors (Affective, Motivational and Cognitive components).

#### First-order factor models

The one-factor model provided a poor goodness-of-fit to the combined data on hope and hopelessness, χ^2^ (350, *n* = 1941) = 14172.30, *p* < .001; CFI = .89; NNFI = .88; RMSEA = .14; Model AIC = 14284.30; Model CAIC = 14652.27. The two-factor model fitted the combined data much better, χ^2^ (349, *n* = 1941) = 6191.10, *p* < .001; CFI = .94; NNFI = .93; RMSEA = .09; Model AIC = 6305.10; Model CAIC = 6679.64, than the one-factor model (*Δ*χ^2^ (1, *n* = 1941) = 7981.20, *p* < .001).

#### Second-order factor models

The single second-order factor model provided an acceptable goodness-of-fit to the hope and hopelessness data, χ^2^ (345, *n* = 1941) = 4686.37, *p* < .001; CFI = .94; NNFI = .94; RMSEA = .08; Model AIC = 4808.37; Model CAIC = 5209.20, whereas the two second-order factor model (Hope and Hopelessness) provided a better goodness-of-fit to the combined data than the one second-order factor model, χ^2^ (344, *n* = 1941) = 3910.08, *p* < .001; CFI = .96; NNFI = .95; RMSEA = .07; Model AIC = 4034.08; Model CAIC = 4441.48. The improvement of fit of the two second-order factor model was statistically significant (*Δ*χ^2^ (1, *n* = 1941) = 776.29, *p* < .001). The two second-order factor model was thus taken as the best-fitted model of hope and hopelessness. The correlation between the two second-order factors was-.61 (*p* < .05), which indicates that hope and hopelessness had a shared variance of about 37%. This is consistent with our hypothesis that hope and hopelessness fitted significantly better to the data as two distinct but correlated factors rather than one unidimensional factor in both first-order and second-order measurement models.

### Negative Binomial Analyses with Hope as Moderator

Three negative binomial regression models were tested. Results of the negative binomial analyses are summarized in Table 3. The first model being tested had hopelessness as the single predictor of suicidal ideation, and the model was significant (Wald *χ* ^2^ (*df* = 1) = 610.32, *p* < .001). Hopelessness was found to be positively correlated with suicidal ideation (*B* = .041, SE = .002; Wald *χ* ^2^ = 534.95, *p* < .001). Hope was then entered in the second model with hopelessness, and the model was significant with a better model fit (*χ* ^2^ (*df* = 2) = 725.36, *p* < .001). Hope was found to be negatively correlated with suicidal ideation (*B* = -.074, SE = .007; Wald *χ* ^2^ = 108.74, *p* < .001). Finally, the interaction term for hope and hopelessness was entered into the third model together with hope and hopelessness, and the model was significant with a better model fit (Wald *χ* ^2^ (*df* = 3) = 729.78, *p* < .001). This shows that hope was a significant moderator for the effect of hopelessness on suicidal ideation.

**Table 3:** Negative Binomial Regression Analyses Predicting Suicidal Ideation.

Model	*B*	SE	Wald *χ* ^2^
Model 1 (*df* = 1)			610.32^a^
Hopelessness	.041	.002	534.95^a^
Model 2 (*df* = 2)			725.36^a^
Hopelessness	.028	.002	178.41^a^
Hope	-.074	.007	108.74^a^
Model 3 (*df* = 3)			729.78^a^
Hopelessness	.028	.002	171.94^a^
Hope	-.070	.007	89.88^a^
Hope × Hopelessness	-.001	.0004	4.23^b^

^a^ *p* < .001

^b^ *p* < .05

**Fig 3:**
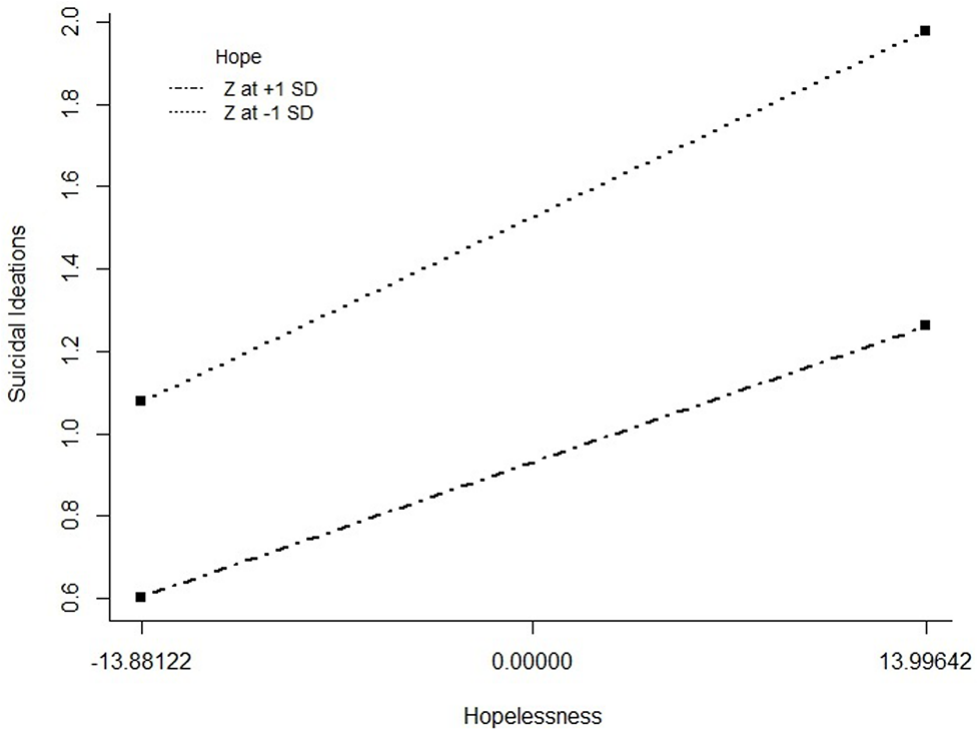
Interaction Plot of Hope Moderating Hopelessness in Predicting Suicidal Ideation. Level of hope (as measured by the Hope Scale) moderates the relationship between hopelessness (as measured by the Beck Hopelessness Scale) and suicidal ideation (as measured by the Adult Suicidal Ideation Questionnaire). For individuals high on hope (scoring one standard deviation above the mean on the Hope Scale), there is smaller increase in suicidal ideation at higher levels of hopelessness than individuals low on hope (scoring one standard deviation below the mean on the Hope Scale).

The deviances of the three negative binomial regression models are shown in Table 4. Based on the deviance analysis, there is improved fit among the three over the previous models, ∆deviance _Model 1—Model 2_ = 115.04, *p* < .001; ∆deviance _Model 3—Model 2_ = 4.42, *p* < .05. Thus, the best model in predicting suicidal ideation is the model with two main effects (hope and hopelessness) and an interaction effect between hope and hopelessness. The interaction between variables is plotted in Fig 3. At low hope (1SD below the mean), the association between hopelessness and suicidal ideation was positive (simple slope = 0.028, *t* = -7.05, *p* < .05). At high hope (1SD above the mean), the association between hopelessness and suicidal ideation was smaller but still positive (simple slope = 0.027, *t* = -6.55, *p* < .05).

**Table 4:** Analysis of Deviance for Negative Binomial Analyses Predicting Suicidal Ideation.

Model	deviance	*df*	∆deviance	∆*df*	*χ* ^2^
Model 1: Hopelessness	4509.91	1931			
Model 2: Hopelessness + Hope	4394.87	1930	115.04	1	115.04^a^
Model 3: Hopelessness + Hope + Hope × Hopelessness	4390.45	1929	4.42	1	4.42^b^

^a^ *p* < .001

^b^ *p* < .05

## Discussion

The present study contributed to the exploration of whether hope and hopelessness constitute a single bipolar construct or two distinct constructs. The empirical findings of the present study suggest that it would be more appropriate to conceive of hope and hopelessness as distinct but related constructs. Although the two constructs shared a portion of their variance (37%), the two second-order factor model (of hope and hopelessness) fitted significantly better to the combined data than the model collapsing the two constructs into a single factor, as hypothesized (*H*1). Moreover, it is noteworthy that the overall hopelessness correlated more strongly (*z* = 3.36, *p* < .001) with the agency component (*r* = -.55) than the pathways component (*r* = -.46) of hope. This observation supports our argument that the hope (esp. the pathways component emphasizing the cognitive process related to the pursuit of a goal as in Snyder’s theory of hope) is distinctive from hopelessness (which is a general negative expectation towards the future). Results consistent with this claim were also observed in the correlation between the pathways component of hope and the cognitive component of hopelessness (*r* = -.36, *p* < .001), which was lower than the other inter-factor correlations. Furthermore, this result is consistent with current findings that agency seems to be a more central component of hope than pathways [66].

It is intriguing that some people choose to end their lives under adversity, while others choose to take their hardships as a challenge and find meaning in their life despite high levels of hopelessness. Here, we propose that hope may be a key factor underlying an individual’s choice to persevere when facing adversity, and test the buffering effect of hope on the association between hopelessness and suicidal ideation using the buffering hypothesis for resilience factors [10]. As hypothesized (*H*2), the moderating effect of hope on the association between hopelessness and suicidal ideation was significant. From the interaction plot (see Fig 3), it can be observed that the level of hope moderates the relationship between hopelessness and suicidal ideation. Those scoring one standard deviation above the mean on hope (high-hope individuals) demonstrated a smaller relationship between hopelessness and suicidal ideation than those scoring one standard deviation below the mean (low-hope individuals). Therefore, hope (as a resilience factor) reduces the negative impact of hopelessness (as a risk factor) on suicidal ideation and buffers individuals against the development of suicidal ideation in the face of hopelessness.

Although there is extensive evidence supporting the relationship between hopelessness and suicidality [43,45], hopelessness may not develop into suicidal ideation if hope is present. Although high-hope individuals may feel depressed, they are likely to rebound from the state of hopelessness when confronting adversity by formulating new goals or redirecting their goals; they may interpret goal blockage as a challenge and seek alternative pathways to re-channel their motivation toward achieving their goals. Conversely, goal blockage in adversity could be disastrous to low-hope individuals as they might fail to generate new goals or find alternative ways to achieve their goals. They may feel hopeless in such a situation and perceive suicide as the only way out, thus resulting in suicidal ideation.

The clarification of hope and hopelessness as separate dimensions, with hope being a resilience factor which can buffer the strength of the association between a risk factor (e.g. hopelessness in the present study) and suicidality (e.g. suicidal ideation in the present study), has implications in future research and interventions in practice. Research in suicidality has mainly focused on identifying risk factors and populations at high risk for suicide. Positive psychological constructs, such as hope, have been largely neglected in the field [67]. In fact, hope is a well-researched construct in other disciplines like sociology and religion. For instance, Stack and Kposowa [68] proposed that a belief in an afterlife of some religions is fundamental to promoting human hopefulness which may lead to lower suicidality. Although the sociological perspective of hope in Stack and Kposowa’s study [68] and our present study are vastly different, the differences and similarities of the construct of hope in different disciplines could be investigated in future.

The findings of the present study suggest that hope can moderate the association between hopelessness and suicidal ideation, and that by instilling and strengthening hope we may be able to lower the risk of suicide. Hope can be further contributed to the design of interventions utilizing the public health approach [69] by using the concept of hope to facilitate the early prevention of suicide and to benefit other public health (e.g., mental health) and social phenomena (e.g., poverty). As stated by Snyder [40], hope theory has the potential for large-scale application and can be used to reduce the risk of, and inoculate segments of society against despair. If community-based prevention programmes involving the promotion of hope are developed, they may help reduce the population at risk. Apart from promoting hope as an early intervention, hope may also be applied in providing timely intervention for those who are at high risk. Pompli [70] called for focusing suicide as a phenomenon affecting unique individuals with unique motives for the suicidal act, and from this we add that the uniqueness of individuals’ level of hope and hopelessness may contribute to the understanding for the phenomenology of suicide and thus clinicians or helpers need to understand patients’ level of hope and hopelessness when working with suicidal individuals. Research has shown that the instillation of positive psychological constructs such as hope [25] and optimism [48,71] in psychotherapy may also be effective for high-risk groups, including suicide attempters and survivors in suicide prevention programmes as well as in depressive patients. Hope training in the form of cognitive behavioral therapy (CBT) which involves the generation of agency and the provision of new pathways to those of high-risk for suicide or depression may be effective in reducing the risk of suicidal intent and depressive symptoms.

The present study has several limitations which need to be considered when generalizing the findings. Firstly, all the variables in present study, including the measures of suicidal ideation (ASIQ), were self-reported. Items of the ASIQ were included in a self-completion questionnaire in order to protect privacy. Secondly, the response rate of the study was only 62%. However, this is not dissatisfactory, as other population-based surveys conducted in Hong Kong on relatively less sensitive topics also obtained similar response rates [72]. Thirdly, the target sample only included individuals aged between 20 and 59 years on account of the design of the ASIQ. Thus, the results of the present study may not be generalizable to other age groups. Future studies should investigate whether the moderating effect of hope on hopelessness and suicidal ideation is also present in samples of the young and elderly. In addition, the study is limited as it utilized a cross-sectional design to investigate correlations, which cannot be used to demonstrate the causal effect of hope in reducing hopelessness and suicidal ideation. Although strict experimental control may not be feasible, future studies should use an experimental approach to examine the effect of different levels of hope on hopelessness and suicidal ideation. For example, one of the authors is conducting a study on examining whether reading a hopeful story enhances a person’s hope, and thus leads to a decrease in hopelessness by priming.

It is important to distinguish and clarify the constructs of hope and hopelessness, that they may be distinct constructs rather than simple correlates or polar opposites of future expectation. Researchers and clinicians need to realize the negative-type constructs are not simply polar opposites of positive-type constructs, that two entities must be considered as unique groups of variables. This is important to the development of the field of positive psychology, as well as the future implication on using positive psychological factors in buffering against negative outcomes.

## Conclusion

In this study, we have explored the constructs of hope and hopelessness using empirical data. Our results have shown that a two second-order factors model of hope and hopelessness fitted better than a single second-order factor combining hope and hopelessness into a single construct. Thus, the results suggested that hope and hopelessness are two distinct but correlated constructs. Hope was further examined as a resilience factor as outlined by the buffering framework. It was found that hope could moderate the negative impact of hopelessness on suicidal ideation. Future research and interventions in practice may act on hope as a resilience factor to study or reduce the negative impacts of suicidality.
